# Exploring the relationship between Treg-mediated risk in COPD and lung cancer through Mendelian randomization analysis and scRNA-seq data integration

**DOI:** 10.1186/s12885-024-12076-1

**Published:** 2024-04-11

**Authors:** Dengfeng Zhang, Haitao Liu, Fangchao Zhao, Pengfei Guo, Jing Li, Tianxing Lu, Zhirong Li, Shujun Li

**Affiliations:** 1 Provincial Center for Clinical Laboratories，Department of Thoracic Surgery, The Second Hospital of Hebei Medical University, Shijiazhuang, China; 2 College of Life Science, Inner Mongolia University, Hohhot, China

**Keywords:** scRNA-seq, Mendelian randomization, Lung cancer, Regulatory T cells, Chronic obstructive Pulmonary disease

## Abstract

**Background:**

Evidence from observational studies suggests an association between chronic obstructive pulmonary disease (COPD) and lung cancer. The potential interactions between the immune system and the lungs may play a causative role in COPD and lung cancer and offer therapeutic prospects. However, the causal association and the immune-mediated mechanisms between COPD and lung cancer remain to be determined.

**Methods:**

We employed a two-sample Mendelian randomization (MR) approach to investigate the causal association between COPD and lung cancer. Additionally, we examined whether immune cell signals were causally related to lung cancer, as well as whether COPD was causally associated with immune cell signals. Furthermore, through two-step Mendelian randomization, we investigated the mediating effects of immune cell signals in the causal association between COPD and lung cancer. Leveraging publicly available genetic data, our analysis included 468,475 individuals of European ancestry with COPD, 492,803 individuals of European ancestry with lung cancer, and 731 immune cell signatures of European ancestry. Additionally, we conducted single-cell transcriptome sequencing analysis on COPD, lung cancer, and control samples to validate our findings.

**Findings:**

We found a causal association between COPD and lung cancer (odds ratio [OR] = 1.63, 95% confidence interval [CI] = 1.31–2.02, *P*-value < 0.001). We also observed a causal association between COPD and regulatory T cells (odds ratio [OR] = 1.19, 95% confidence interval [CI] = 1.01–1.40, *P*-value < 0.05), as well as a causal association between regulatory T cells and lung cancer (odds ratio [OR] = 1.02, 95% confidence interval [CI] = 1.002–1.045, *P*-value < 0.05). Furthermore, our two-step Mendelian randomization analysis demonstrated that COPD is associated with lung cancer through the mediation of regulatory T cells. These findings were further validated through single-cell sequencing analysis, confirming the mediating role of regulatory T cells in the association between COPD and lung cancer.

**Interpretation:**

As far as we are aware, we are the first to combine single-celled immune cell data with two-sample Mendelian randomization. Our analysis indicates a causal association between COPD and lung cancer, with regulatory T cells playing an intermediary role.

**Supplementary Information:**

The online version contains supplementary material available at 10.1186/s12885-024-12076-1.

## Introduction

Chronic obstructive pulmonary disease (COPD) and lung cancer are the two respiratory diseases with the highest mortality rates globally [1]. There is a significant comorbidity relationship between COPD and lung cancer, with COPD patients having a markedly higher risk of developing lung cancer compared to the general population [2]. Relevant studies have shown that the relationship between COPD and lung cancer may originate from the chronic inflammatory state caused by COPD itself [3], as well as the resultant immune dysfunction [4, 5].

In recent years, Mendelian randomization studies have been widely used to explore causal relationships between complex diseases [6, 7]. As a genetic matching approach, it can effectively control for confounding from environmental and behavioral factors, providing more reliable causal inference. However, this method has not been extensively adopted in research on the relationship between COPD and lung cancer. Additionally, advances in single-cell sequencing technologies have enabled in-depth profiling of various cell subpopulations [8]. This technique has seen widespread application in cancer research, revealing the important roles of immune cells in tumor initiation and progression. Nevertheless, there have been fewer studies incorporating immune factors to investigate the link between COPD and lung cancer thus far. Regulatory T cells (Tregs) are thought to be involved in the pathogenesis of both COPD and lung cancer. Tregs can suppress immune responses, and their excessive activation has been associated with development and poor prognosis of COPD and lung cancer [9, 10]. However, the causal relationship between COPD and lung cancer as well as the mechanistic role of Tregs remains unclear.

In the present study, we utilized Mendelian randomization to explore the causal relationship between these two complex diseases [11]. Additionally, we leveraged single-cell sequencing technology to analyze infiltration and expression patterns of different cell types at the single-cell resolution [12]. Our study provides an in-depth investigation into the causal link between COPD and lung cancer as well as the mediating role of Tregs, laying a theoretical foundation concerning the connection between the two respiratory illnesses and immunotherapies.

## Methods

### Immunity-wide GWAS data

The summary statistics for each immunophenotype can be publicly accessed from the GWAS Catalog (accession numbers from GCST90001391 to GCST90002121, encompassing a total of 731 immunophenotypes, including absolute cell (AC) counts (*n* = 118), median fluorescence intensity (MFI) reflecting surface antigen levels (*n* = 389), morphological parameters (MP) (*n* = 32), and relative cell (RC) counts (*n* = 192). These features encompass various developmental stages and cell types of immune cells. The original GWAS for immunophenotypes utilized data from 3,757 European individuals, with non-overlapping cohorts. The significance level for instrumental variables (IV) for each immunophenotype was set at 1 × 10^(-5). We pruned these SNPs (linkage disequilibrium [LD] r^2^ threshold of < 0.1 within a 500 kb distance).

### Lung cancer and COPD GWAS data

The lung cancer (*n* = 492,803) and COPD (*n* = 468,475) GWAS data were obtained from the GWAS Catalog (accession numbers ebi-a-GCST90018875 and ebi-a-GCST90018807). The significance level for each instrumental variable (IV) was set at 5 × 10^(-8). We pruned these SNPs (linkage disequilibrium [LD] r^2^ threshold of < 0.001 within a 10,000 kb distance)

### **scRNA-seq data**

The scRNA-seq data of human lung cancer patients were downloaded from the GEO database with the accession number: GSE131907 and GSE173896. We utilized the Seurat R package to identify distinct cell types and examine variations in immune cell infiltration. Cells that met specific criteria, such as having fewer than 200 genes, over 5,000 genes, or more than 20% mitochondrial expression, were excluded from the analysis. Raw counts were normalized using the ‘NormalizeData’ function, and variable genes were identified using the ‘FindVariableGenes’ function. Subsequently, dataset expression values were scaled and centered using the ‘ScaleData’ function to reduce dimensionality. Principal component analysis (PCA) and the uniform manifold approximation and projection (UMAP) methods were employed to visualize the data in lower dimensions, with the first two dimensions chosen for plotting. Cell clustering was performed using the ‘FindClusters’ function, and highly expressed genes within each cell cluster were determined using the ‘FindAllMarkers’ function. Additionally, the ‘FindMarkers’ function was used to identify differentially expressed genes (DEGs) between two cell populations.

### Statistical analysis

Our Mendelian randomization (MR) analysis was primarily conducted using the inverse variance-weighted (IVW) method with the R package TwoSampleMR. To account for the potential influence of horizontal pleiotropy, we employed a commonly used method, MR-Egger, which identifies the presence of horizontal pleiotropy if its intercept term is significant. Heterogeneity in the effect size of SNP-specific causal effects during two-sample MR was assessed using Cochran’s Q-test. Finally, we performed a leave-one-out sensitivity analysis to evaluate the impact of individual SNPs on the overall estimates. We defined the presence of heterogeneity when the *P*-value for Cochran’s Q-statistic was less than 0.05. Although heterogeneity was detected, it did not impact the results of the IVW analysis, and our conclusions remain reliable. We incorporated a Colorful-clouds two-stepMR analysis to validate the mediating effect of regulatory T cells in the association between COPD and lung cancer. The bioinformatics analysis is done through R software and bioinformatics tools.

## Results

### The discussion of the causal effect between COPD and lung cancer

**Fig. 1:**
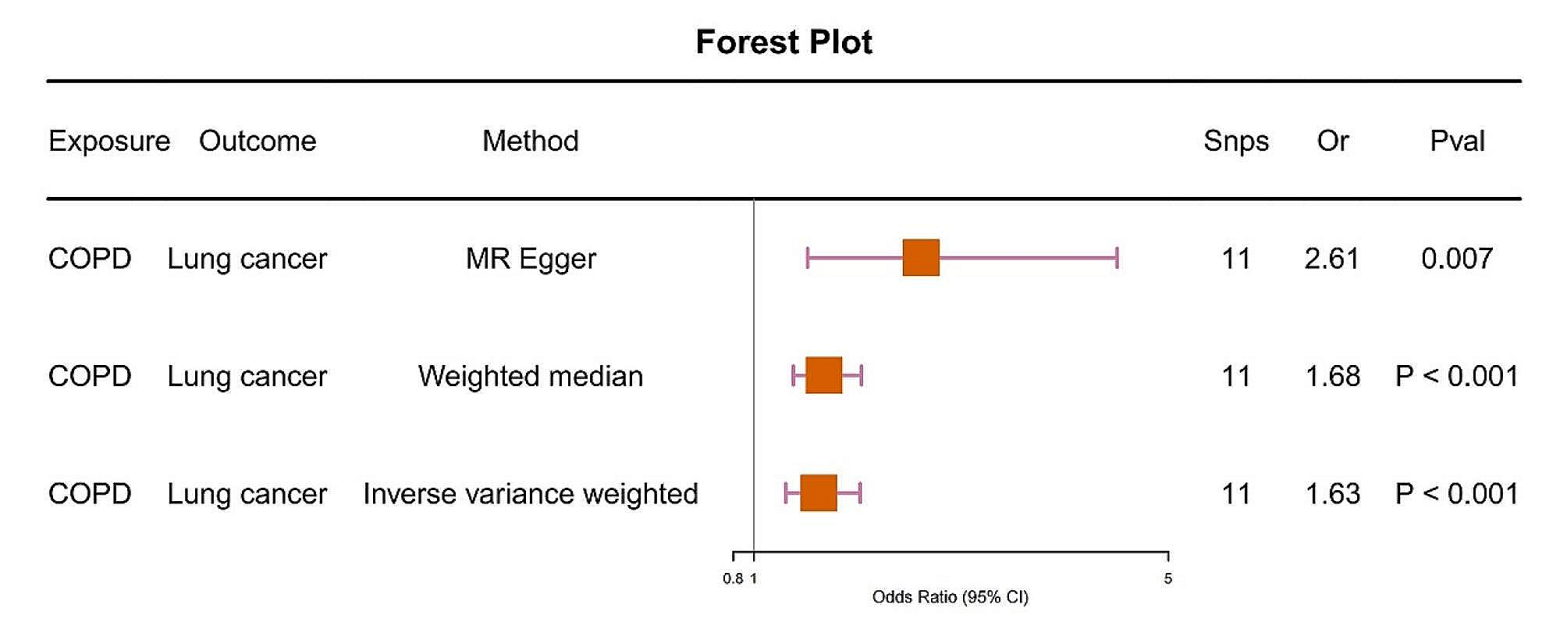
Using various methods to analyze the causal association between COPD and lung cancer

We assessed whether COPD is associated with lung cancer causally. We primarily used the IVW method, followed by MR Egger and Weighted median methods. All three methods consistently showed that COPD is causally associated with lung cancer, with an odds ratio (OR) of 1.63 and a 95% confidence interval (CI) of 1.31–2.02, and a *P*-value < 0.001 (Fig. 1). We validated the reliability of our results through horizontal pleiotropy tests, heterogeneity tests, and Leave-One-Out (LOO) analysis. We conducted a test for horizontal pleiotropy using the MR Egger method. The *P*-value for the MR-Egger regression intercept was 0.099, which is greater than 0.05, indicating that COPD does not exhibit horizontal pleiotropy (Table S1). The LOO analysis revealed consistent trends for all included SNPs in our analysis, and the scatter plots further demonstrated the robustness of our findings (Fig. S1, 2). Although our heterogeneity tests yielded a *p*-value of less than 0.05, indicating the presence of heterogeneity, it did not impact the results of the Inverse Variance Weighted (IVW) analysis. Therefore, our analysis results remain reliable (Table S2).

### The discussion of the causal effect between COPD and immunophenotypes

**Fig. 2:**
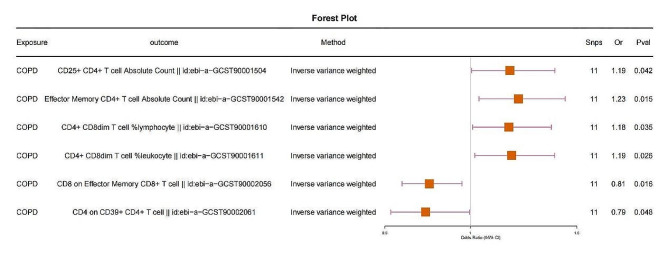
The causal association between COPD and six immune cell phenotypes

To investigate the causal association between immune cells and lung cancer, we conducted a Mendelian randomization study with 731 immune cell phenotypes as exposures and lung cancer as the outcome. The results revealed that 51 immune cell phenotypes are causally related to lung cancer (*p*-value < 0.05) (Fig. S3 and Table S3). Furthermore, using these 51 immune cell phenotypes as outcomes and COPD as the exposure, we explored whether these immune cells could potentially act as intermediate factors mediating the causal association between COPD and lung cancer. The findings indicate that COPD is causally related to CD25 + CD4 + T cell Absolute Count (Treg, odds ratio [OR] = 1.19, 95% confidence interval [CI] = 1.01–1.39, *P*-value < 0.05), CD8 on Effector Memory CD8 + T cell (CD8 + T cell, odds ratio [OR] = 0.81, 95% confidence interval [CI] = 0.68–0.96, *P*-value < 0.05), and CD4 + CD8dim T cell %lymphocyte (CD4 + T cell, odds ratio [OR] = 1.19, 95% confidence interval [CI] = 1.02–1.39, *P*-value < 0.05) (Fig. 2). We validated the reliability of our results through horizontal pleiotropy tests and heterogeneity analyses. The results demonstrated *p*-values all greater than 0.05, indicating the absence of heterogeneity and horizontal pleiotropy between COPD and these immune cell phenotypes (Table S4, 5). This suggests that our analytical findings are reliable. These results indicate that COPD exhibits different directional causal associations in CD4 + T cells (OR > 1) and CD8 + T cells (OR < 1) (Fig. 2).

### Exploring the infiltration dynamics of T cells in COPD, lung cancer, and normal lungs

**Fig. 3:**
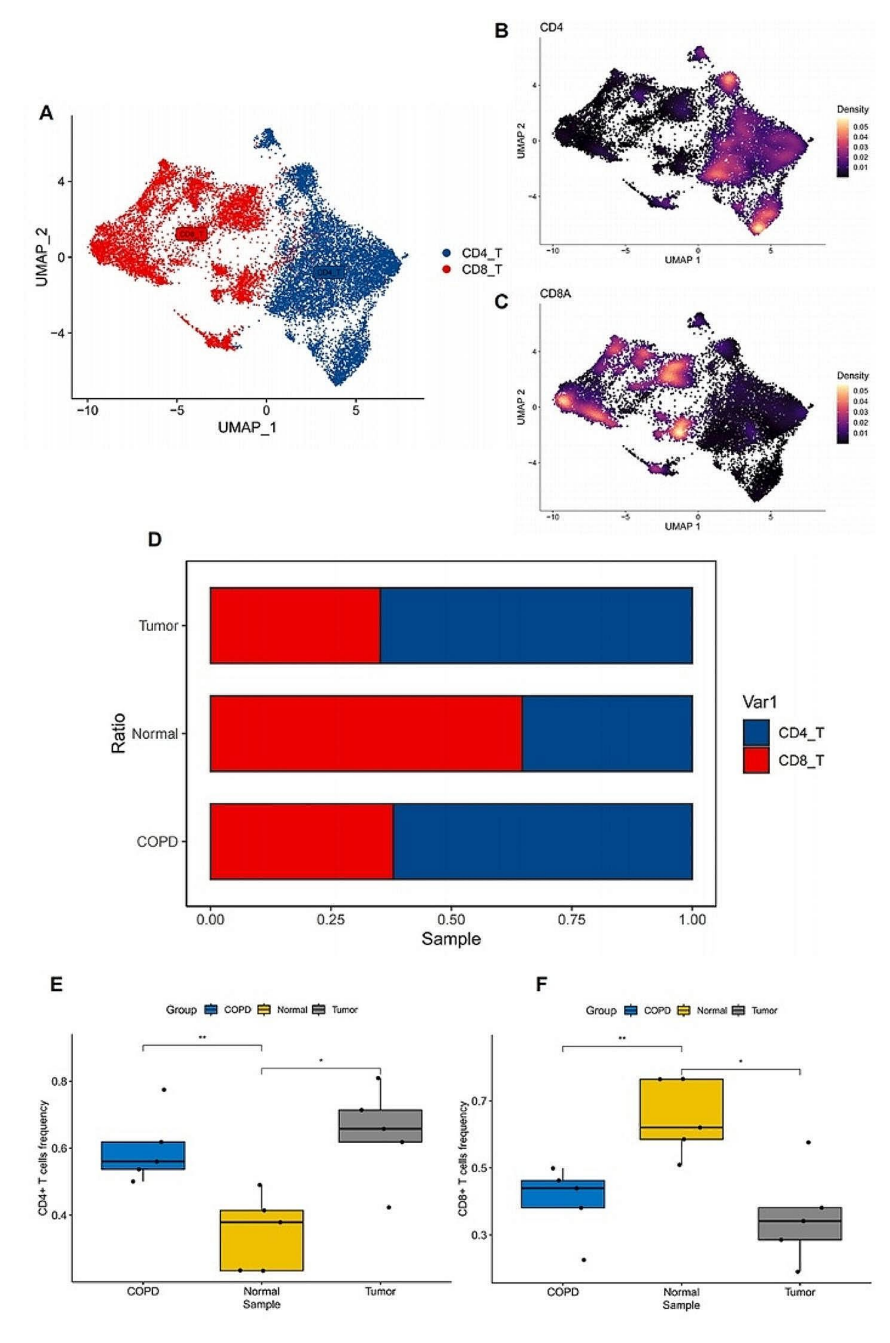
Single-cell sequencing reveals the dynamic changes of CD4 + T cells and CD8 + T cells in COPD and lung cancer. (**A**) UMAP plot showing the subtypes of T cells, including CD4 + T cells and CD8 + T cells. (**B** and **C**) The expression pattern of CD4 and CD8A markers in T cell subpopulations. (**D**) Stacked barplot comparing the relative frequencies of CD4 + T cells and CD8 + T cells among COPD, lung cancer and normal samples. (**E** and **F**) Boxplots showing the distribution of frequencies for CD4 + T cells and CD8 + T cells across different sample groups

To further investigate the infiltration of T cells in COPD, lung cancer, and normal lung tissues, we reanalyzed single-cell data obtained from the lungs of 15 patients, including 5 with COPD, 5 with lung cancer, and 5 with normal lungs. To ensure the selection of high-quality T cells, we performed a subset operation on the single-cell data (CD3D > 0| CD3E > 0| CD3G > 0), ensuring that the extracted data exclusively represented T cells. Ultimately, we obtained 17,133 high-quality T cells from these 15 samples. Using uniform manifold approximation and projection (UMAP) based on the expression of CD4 and CD8A, we visualized the resulting data, categorizing T cells into CD4 + T cells and CD8 + T cells (Fig. 3A, B, C). Further observation of the infiltration patterns of CD4 + and CD8 + T cells revealed a consistent trend in COPD and lung cancer. Specifically, CD4 + T cells were significantly elevated, while CD8 + T cells were significantly decreased compared to normal lung tissues. Consistent with our Mendelian randomization analysis, CD4 + T cells and CD8 + T cells exhibited different directional trends. Moreover, in comparison to COPD, lung cancer demonstrated higher infiltration of CD4 + T cells and lower infiltration of CD8 + T cells (Fig. 3D, E, F).

### Exploring changes in CD4 T cell subtypes and their role in COPD and lung cancer

**Fig. 4:**
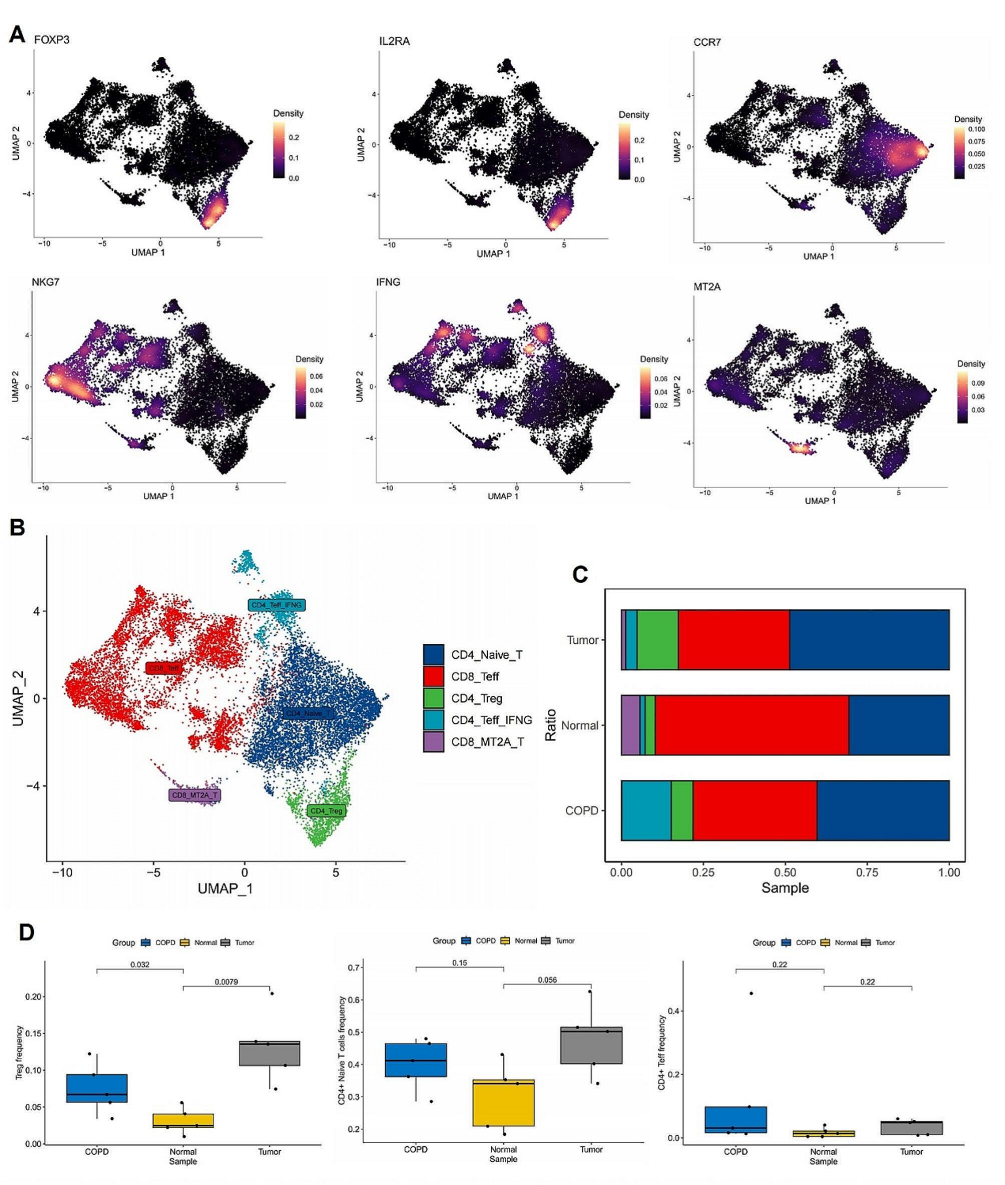
Single-cell sequencing reveals the dynamic changes of T cell subtypes in COPD and lung cancer. (**A**) The expression of marker genes in T cells. (**B**) UMAP plot showing the subtypes of T cells. (**C**) Stacked barplot comparing the relative frequencies of each T cell subtype among COPD, lung cancer and normal samples. (**D**) Boxplots showing the distribution of frequencies for each T cell subtype across different sample groups

To investigate which T cell subtypes undergo changes within CD4 T cells, we performed subtype annotation on T cells. The results revealed CD4 + naive T cells (CD4_Naive_T, CCR7), CD8 + effector T cells (CD8_Teff, NKG7), regulatory T cells (Treg, FOXP3 and IL2RA), CD4 + effector T cells (CD4_Teff_IFNG, IFNG), and T cells with high expression of MT2A (CD8_MT2A_T) (Fig. 4A, B). We further observed changes in their infiltration, and the results showed that, although COPD and lung cancer exhibited increases in various CD4 T cell subtypes compared to normal lungs, only Treg cells showed statistical significance (*p*-value < 0.05) (Fig. 4C, D). Consistent with our previous Mendelian randomization analysis, a two-step method confirmed the causal effect of COPD on Treg and the causal effect of Treg on lung cancer (Fig. S3 and Table S7). The odds ratio (OR) values were consistently greater than 1, suggesting that Treg serves as an intermediate factor mediating the causal relationship between COPD and lung cancer. Further support from our single-cell sequencing analysis substantiates Treg’s role as an intermediate factor in mediating the causal relationship between COPD and lung cancer.The list of SNPs/genes used as instrumental variables is stored in Table S6. 

To further validate our results and ensure the robustness of the findings, we conducted multivariable Mendelian randomization analysis, considering smoking as an important confounding factor. The results showed that even when considering smoking as a confounder, COPD still had a causal relationship with lung cancer (Fig. S4). Then, we conducted bidirectional Mendelian randomization analyses for COPD and lung cancer, as well as immune cell phenotypes and COPD. The purpose of this analysis was to validate the association results we observed earlier to ensure the reliability of our study findings. The results showed no causal effects of COPD or lung cancer on immune cell phenotypes (Fig. S5), nor did immune cell phenotypes have a causal effect on COPD (Fig. S6). This further supports our main findings and enhances our confidence in the causal relationships between genes and diseases.

## Discussion

We identified a causal association between the genetic liability to Treg infiltration and lung cancer, as well as a causal association between the genetic liability to COPD and Treg infiltration. Further validation through single-cell transcriptome sequencing analysis and multivariable Mendelian randomization confirmed Treg cells as intermediate factors in the causal association between COPD and lung cancer. Multiple studies have demonstrated a correlation between chronic obstructive pulmonary disease (COPD) and lung cancer, with COPD patients exhibiting a markedly higher risk for developing lung cancer compared to the general population [13–15]. Cigarette smoking is a shared risk factor for both COPD and lung cancer [16]. Additionally, the chronic inflammatory state induced by COPD itself also increases the likelihood of lung carcinogenesis [17], through several potential mechanisms: (1) COPD-associated chronic inflammation stimulates tumor emergence; (2) COPD destroys alveolar architecture, enabling easier access of carcinogens into the lungs; (3) COPD impairs systemic immunosurveillance, heightening possibilities for malignant transformation. The close association between the development of COPD and lung cancer has been extensively reported, with numerous studies suggesting that the progression of COPD may contribute to the occurrence of lung cancer. Our Mendelian randomization analysis provided causal evidence supporting this association between COPD and lung cancer.

The initiation and progression of lung cancer is profoundly impacted by the modulation of immune cells within the tumor microenvironment. At different stages of lung carcinogenesis, fluctuations in the abundance and functional status of various immune cell subsets lead to dampened immune surveillance and cytotoxicity against malignant cells, thereby fostering tumor growth and progression. Specifically, decreased tumor-infiltrating CD8 + cytotoxic T lymphocytes in the lung cancer microenvironment fail to effectively contain tumor expansion [18]. In contrast, increased proportions of tumor-associated macrophages [19], regulatory T cells [20], tumor-associated neutrophils [21], and other cell types assist tumor immune evasion through angiogenesis promotion, immunosuppressive factor secretion, and related mechanisms, conferring survival and proliferative advantages. Additionally, diminished natural killer cell activity weakens cytotoxic responses against lung cancer [22].

Prior to our study, there was a lack of research predicting the association between immune cells and the occurrence of lung cancer from a genetic perspective. Through Mendelian randomization analysis, we identified 51 immune cell phenotypes with causal associations with lung cancer. CD4 + and CD8 + T cell subsets play vital roles in the pathological process of COPD. The proportions of CD8 + T cells are elevated in the peripheral blood and airway inflammation of COPD patients, positively correlating with the severity of airway limitation and disease progression [23, 24]. CD8 + T cells can directly kill alveolar and airway epithelial cells, releasing proinflammatory cytokines like TNF-α and resulting in airway and lung tissue damage [25]. In contrast, CD4 + T lymphocytes mainly participate in COPD pathology by secreting cytokines including IL-17 and IFN-γ [26]. In summary, the major pathogenic effect of CD8 + T cells is direct cytotoxicity against structural cells, whereas CD4 + T lymphocytes contribute to COPD development via release of inflammatory mediators.

Our findings demonstrated an increase in CD4 + T lymphocytes along with a decrease in CD8 + T lymphocytes among patients with chronic obstructive pulmonary disease (COPD). This is consistent with existing literature reports, such as the study by Barcelo et al. showing elevated peripheral blood CD4 + T cell percentages combined with reduced CD8 + T cell proportions in COPD patients [9].We observed a causal association between COPD and both CD4 + and CD8 + T cells. Subsequent validation through single-cell sequencing analysis confirmed that the development of COPD is accompanied by an increase in CD4 + T cell infiltration and a decrease in CD8 + T cell infiltration. Multiple previous studies have demonstrated that regulatory T cells (Tregs) play significant roles in the pathogenesis of both COPD and lung cancer. The quantity of Tregs is elevated in the peripheral blood and bronchoalveolar lavage fluid (BALF) of COPD patients, positively correlating with the degree of pulmonary function impairment [27, 28]. Tregs can inhibit effector T lymphocyte activity and mitigate inflammatory responses in lung tissue, but excessive long-term activation may also lead to increased risks of bacterial and viral infections [29]. In lung cancer, higher infiltrative proportions of Tregs in tumor tissue suppress anti-tumor immune responses and associate with poor prognosis [20]. Within the subtypes of CD4 + T cells, we found a significant increase in Treg cell infiltration in both COPD and lung cancer, with Treg cells acting as intermediate factors mediating the causal association between COPD and lung cancer.

Several limitations exist in our study. Firstly, the GWAS data were derived from European databases, representing only the European population and not encompassing all ethnicities. Secondly, our single-cell data were based on a small sample size of 15 patients, and further validation with a larger dataset is warranted.

In conclusion, our analysis demonstrates that Treg cells act as intermediate factors in mediating the causal association between COPD and lung cancer. This provides important insights for the prevention of lung cancer in COPD patients in the future.

### Electronic supplementary material

Below is the link to the electronic supplementary material.

Supplementary Material 1

Supplementary Material 2

## Data Availability

The original datasets analyzed in this study are publicly available through the following repositories: The GWAS data were obtained from the GWAS Catalog and NCBI Gene Expression Omnibus (https://www.ncbi.nlm.nih.gov/gds/). Further inquiries can be directed to the corresponding authors.
