# Supplementary material for: Exploring the relationship between Treg-mediated risk in COPD and lung cancer through Mendelian randomization analysis and scRNA-seq data integration
Source: BMC Cancer. 2024 Apr 11;24:453. doi: 10.1186/s12885-024-12076-1 (PMC11010300; doi:10.1186/s12885-024-12076-1)

**Fig.S1** Scatter plots were employed to validate the robustness of the causal linkage between COPD and lung cancer.


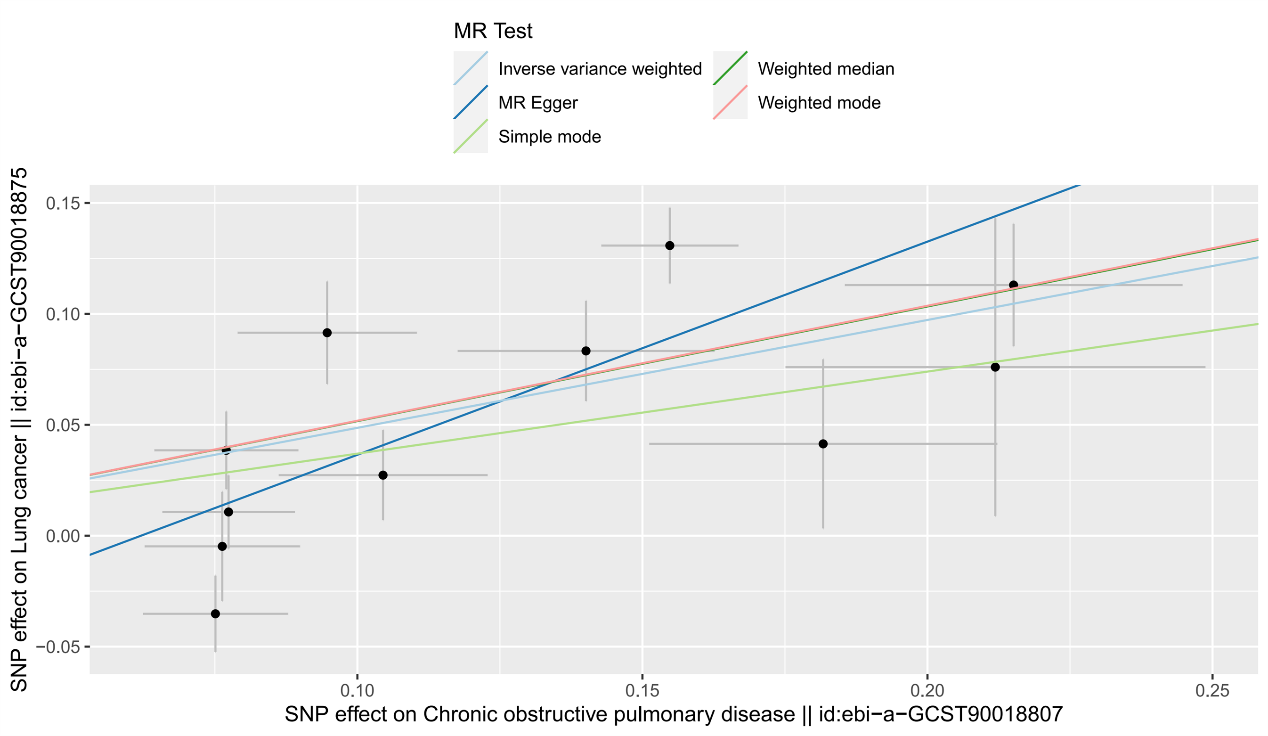


**Fig.S2** Leave-One-Out analysis was performed to authenticate the robustness of the causal relationship between COPD and lung cancer.


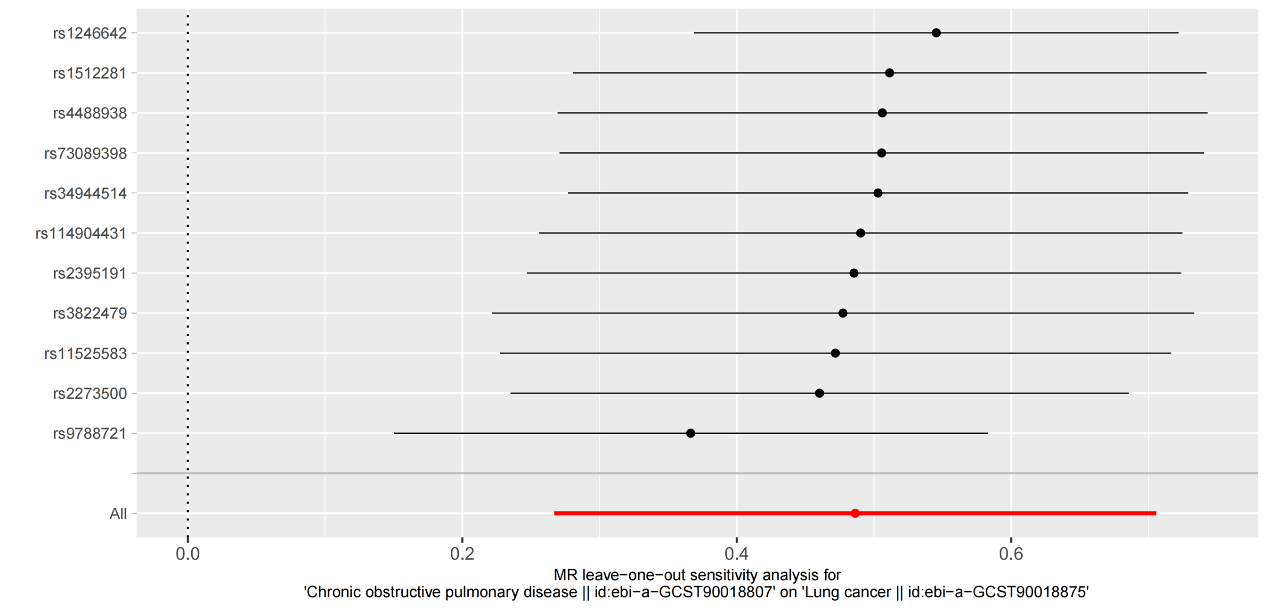


**Fig.S3** Mendelian randomization analysis was conducted to ascertain the causal relationship between immune phenotype and lung cancer.


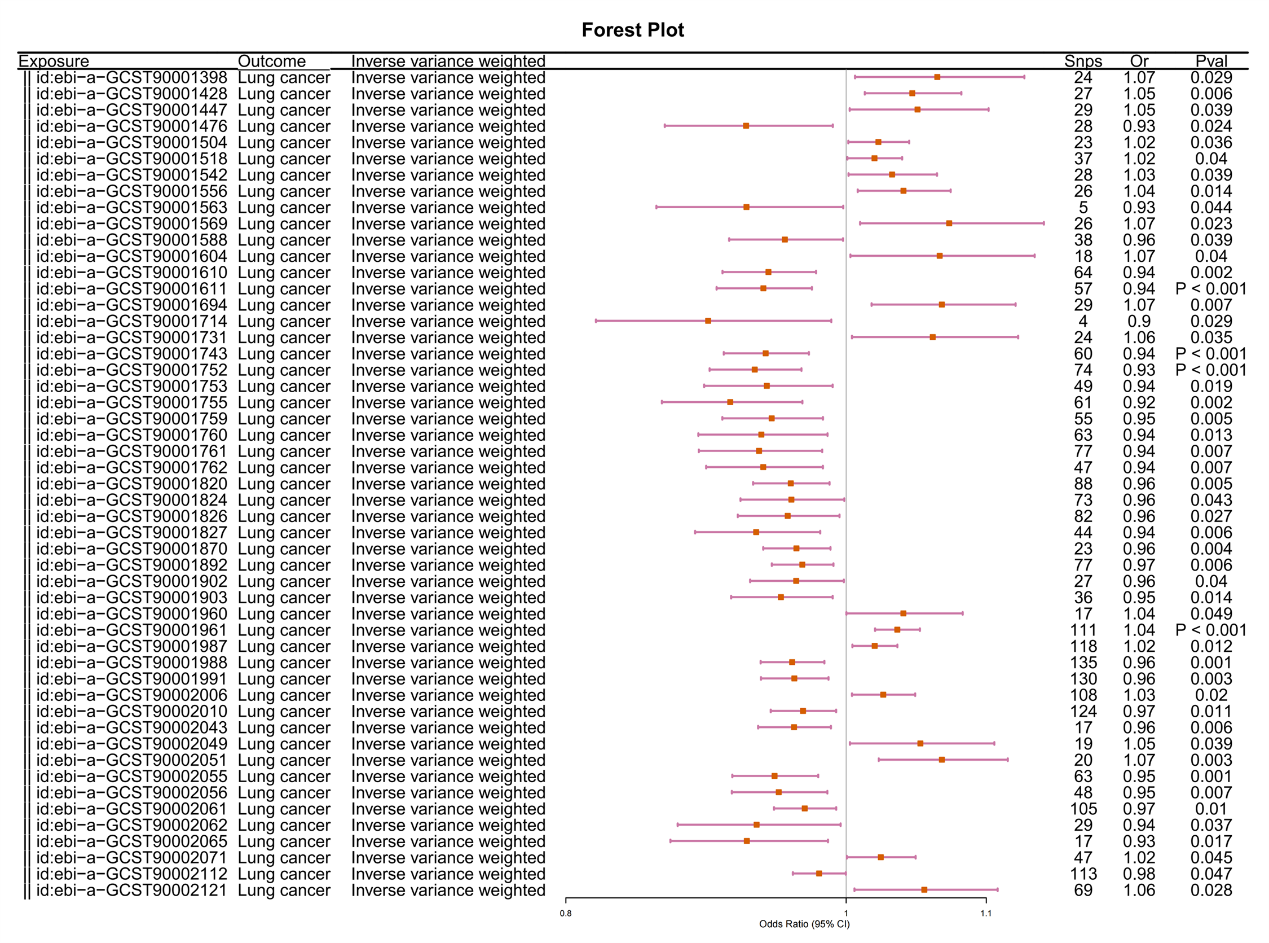


**Fig.S4** Multivariable Mendelian randomization analysis was conducted to investigate the causal relationship between COPD, smoking, and lung cancer.


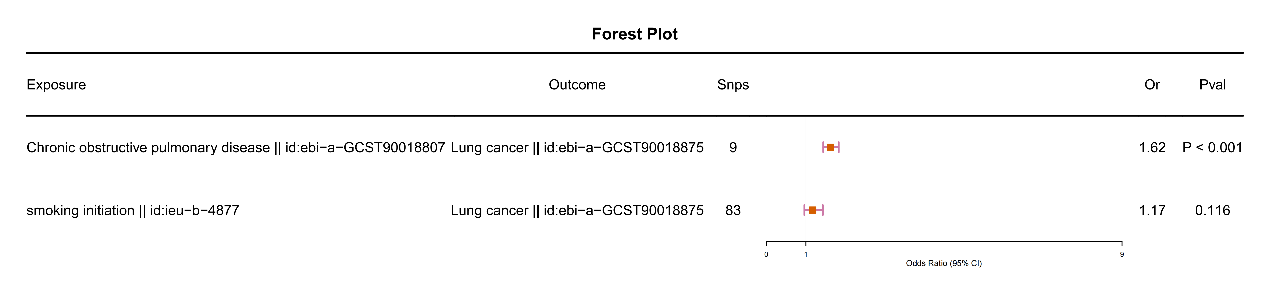


**Fig.S5** Reverse Mendelian randomization analysis was employed to assess the causal relationship between lung cancer as an exposure and COPD as an outcome.


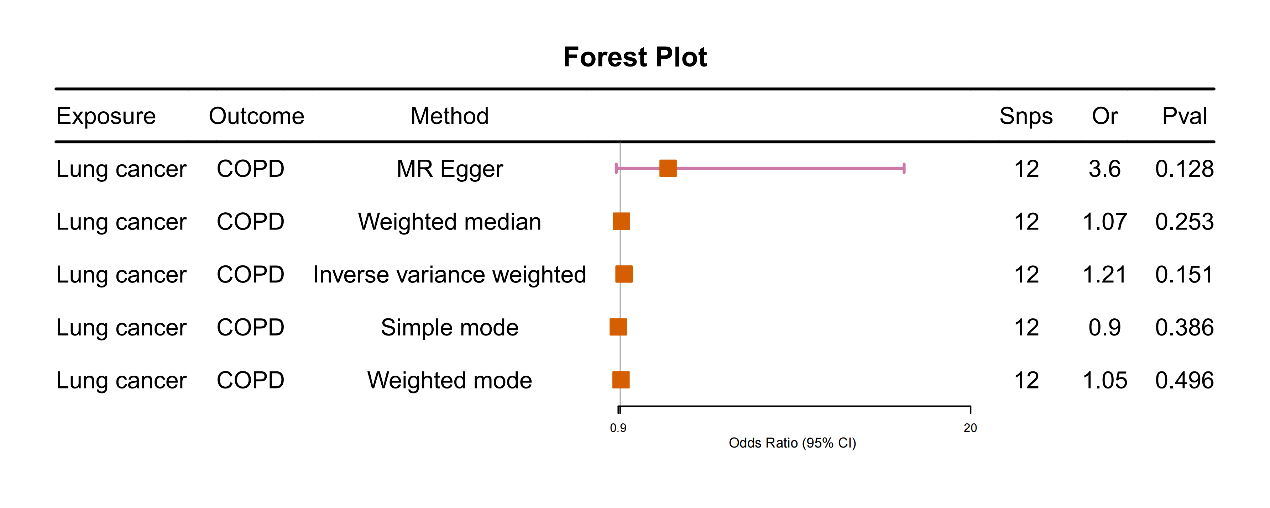


**Fig.S6** Reverse Mendelian randomization analysis was utilized to investigate the causal relationship between Treg as an exposure and COPD as an outcome.


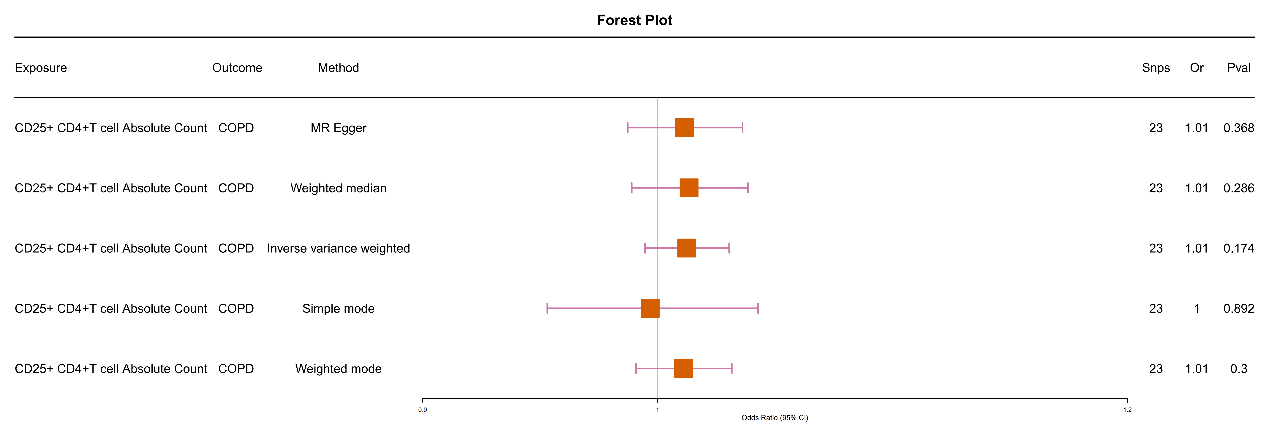

Supplement: Supplementary file 1 — Supplementary Material 1 [file 12885_2024_12076_MOESM1_ESM.docx]
